# The Relationship between *VDR* Gene Polymorphisms *Bsm1* and *Apa1* with Breast Cancer Risk

**DOI:** 10.1055/s-0044-1779040

**Published:** 2024-03-04

**Authors:** Hengameh Mozaffarizadeh, Fariborz Mokarian, Mansoor Salehi, Seyyed Mohammad Reza Hakimian, Elham Moazam, Amirmohammad Amoozadehsamakoosh, Majid Hosseinzadeh, Mahdieh Behnam, Mohaddeseh Behjati, Alma Naseri, Marzieh Lotfi, Fatemeh Tohidi

**Affiliations:** 1 Department of Genetics, Isfahan University of Medical Sciences, Isfahan, Iran; 2 Department of Clinical Oncology, Isfahan University of Medical Sciences, Isfahan, Iran; 3 Department of Clinical Oncology, Cancer Prevention Research Center, Isfahan University of Medical Sciences, Isfahan, Iran; 4 Faculty of Medicine, Babol University of Medical Sciences, Babol, Iran; 5 Department of Medicine, Rajaie Cardiovascular Medical and Research Center, Iran University of Medical Sciences, Tehran, Iran; 6 Department of Medical Biotechnology, Faculty of Medicine, Babol University of Medical Sciences, Babol, Iran; 7 Department of Medical Genetics, Abortion Research Center, Reproductive Sciences Institute, School of Medicine, Shahid Sadoughi University of Medical Sciences and Health Services, Yazd, Iran; 8 Cellular and Molecular Biology Research Center, Health Research Institute, Babol University of Medical Sciences, Babol, Iran; 9 Department of Medical Biotechnology, Cancer Research Center, School of Medicine, Babol University of Medical Sciences, Babol, Iran

**Keywords:** breast cancer, *VDR* gene, polymorphism

## Abstract

**Background** In addition to its multifaceted physiological functions, vitamin D is recognized for its protective role against cancer. To manifest its effects, vitamin D engages with the vitamin D receptor ( *VDR* ) gene responsible for its encoding. Investigations have unveiled that polymorphisms within the *VDR* gene exert influence over the expression and/or functionality of the VDR protein. Notably, certain *VDR* gene polymorphisms have emerged as particularly pertinent in the context of tumorigenesis, including Fok1 (rs2228570), Bsm1 (rs1544410), Taq1 (rs771236), and Apa1 (rs7975232). This study aims to scrutinize the correlation between the Bsm1 and Apa1 polymorphisms and the susceptibility to breast cancer development.

**Materials and Methods** In this study, 50 patients suffering from breast cancer with less than 6 months breast cancer diagnosis and 50 healthy control individuals have been chosen. Restriction fragment length polymorphism polymerase chain reaction was used to determine the genotype of polymorphisms.

**Results** The results of the statistical analysis showed that among the studied polymorphisms, there was no correlation with the development of breast cancer.

**Conclusion** Studies on various cancers have produced inconsistent results regarding vitamin D's role in the development and progression of cancer. Therefore, further research is necessary to determine vitamin D's role in cancer development and progression.

## Introduction

Women globally exhibit a heightened susceptibility to breast cancer, ranking as the second leading cause of mortality following lung cancer. 1 The individual risk of breast cancer manifestation is contingent on a myriad of factors, encompassing age, gender, the presence of benign breast tumors, timing of menopause, hormone therapy, chest radiation exposure, combined use of estrogen and progesterone, alcohol consumption, diethylstilbestrol ingestion, genetic predisposition, postmenopausal obesity, age at first pregnancy exceeding 30 years, breastfeeding practices, and assorted environmental influences. 2 3 4 Breast cancer pathogenesis is intricately linked to genetic determinants, involving disruptions in gene expression levels, epigenetic alterations, and polymorphisms in DNA sequences. 5 Amidst the multifaceted array of environmental factors influencing cancer progression, vitamin D emerges as a significant modulator.

**Fig. 1:**
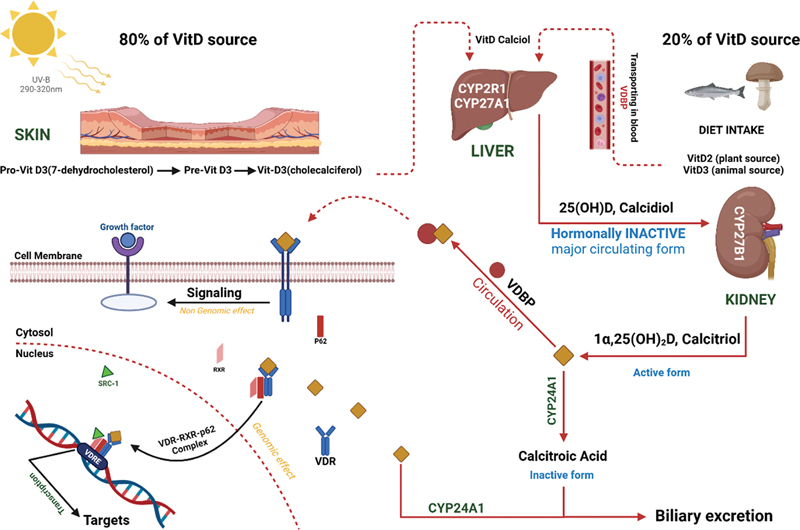
The process of synthesizing calcitriol involves site-specific modifications of 7-dehydrocholesterol in the skin, liver, and kidney. This results in the generation of a bioactive compound that, in conjunction with the vitamin D receptor (VDR), translocates into the cell cytosol through specialized channels. Upon heterodimerization, the ensuing complex translocates into the nucleus, forming a phosphorylated calcitriol–VDR complex alongside the retinol X receptor and 9cRa transcription factor. This multifaceted complex binds to DNA, suppressing the *CYP27B1* gene, which is accountable for parathormone production, in the presence of HDAC complexes and other transcription factors. In contrast, the PBA/SWI/SNF complex facilitates the incorporation of regulatory elements, transcriptional factor IIB, and, notably, RNA polymerase II. This orchestrated process induces the transcription of the *CYP27B1* gene, and the resultant protein, localized to the inner mitochondrial membrane, hydroxylates 25-hydroxyvitamin D3 at the 1α position, culminating in the synthesis of the active form, 1α,25-dihydroxyvitamin D3, which subsequently binds to the VDR.

Occurring in two distinct natural forms—vitamin D2 (ergocalciferol), derived from plant sources, and vitamin D3 (cholecalciferol), synthesized in the dermal layers of humans and animals—vitamin D is predominantly obtained through sunlight exposure (constituting up to 90% of its intake) and dietary supplements. 6 Here are several enzymatic steps and genes involved in the metabolism of vitamin D. 7 The metabolic transformation of vitamin D involves several enzymatic steps, commencing with hepatic conversion yielding 25(OH)D or calcidiol, followed by renal processing resulting in the biologically active form, 1,25(OH)2D or calcitriol ( Fig. 1 ). 8 Beyond its fundamental role in bone metabolism and hemostasis of calcium and phosphorus, vitamin D assumes a pivotal role as a protective factor against cancer across diverse anatomical sites. This protective function is executed through the regulation of gene expression, mitigation of invasiveness, angiogenesis, and modulation of proliferation, differentiation, and apoptosis within various cancerous cell lines. 9 10 11 12 13 14 15 16 17 18 19 20 21 22 23 This regulatory process is enacted through the interaction of vitamin D with its receptor, namely the vitamin D receptor (VDR), which is expressed in more than 30 tissues across the human body. 24 25 Positioned on the long arm of chromosome 12 (12q12–14), the *VDR* gene encompasses a minimum of five promoter regions and at least 11 exons, covering a genomic span of 60 kb. Notably, the initial exon remains untranslated, while exons 2 to 8 encode the VDR protein. 26 27

Numerous studies have substantiated that polymorphisms within the *VDR* gene exert influence over the expression and functionality of the VDR protein. 28 Notably, of the ∼200 VDR polymorphisms documented, Fok1, Bsm1, Taq1, Apa1, EcoRV, and Cdx2 have demonstrated recurrent associations with tumorigenesis, albeit the existing data in this realm exhibit frequent contradictions and necessitate further elucidation. 29 30 Both laboratory inquiries and epidemiological investigations suggest a conceivable correlation between vitamin D levels, the expression of the VDR, and an increased susceptibility to breast cancer. 31 32

*Polymorphism Bsm1* : Polymorphism Bsm1, situated in intron 8, involves the conversion of guanosine to adenosine. It has been postulated that this polymorphism may correlate with poly-A sequences in the 3′ untranslated region (UTR) region, potentially influencing the expression of *VDR* genes by modulating mRNA stability. 33

*Polymorphism Apa1* : Polymorphism Apa1, situated within intron 8, entails the substitution of a thymine nucleotide with guanine. Analogous to the Bsm1 polymorphism, this genetic variation exerts an impact on the expression levels of the VDR protein. 29

The present investigation centers on elucidating the prevalence and distribution patterns of VDR Bsm1 and Apa1 polymorphisms among breast cancer patients in Isfahan, comparing these profiles with those observed in healthy population cohorts.

## Materials and Methods

### Study Population

A case–control investigation was conducted to elucidate the potential link between breast cancer susceptibility and four polymorphisms within the *VDR* gene. A cohort comprising breast cancer patients ( *n* = 50) was systematically selected from individuals referred to three separate breast cancer clinics: Asqarieh Hospital, Al Zahra Hospital, and Ordibehesht Clinic. Standardized diagnostic criteria and management protocols were uniformly applied across these centers, adhering to established international guidelines. Comprehensive patient information, encompassing age, menopausal status, and familial or sporadic background, was systematically collected.

Concurrently, a control group comprised 50 healthy individuals, selected based on their absence of family history pertaining to breast cancer. These controls were drawn from women availing themselves of state-run health care services for routine mother and infant examinations. Ethical considerations were paramount, with each participating patient providing informed consent through the endorsement of a written consent form, duly sanctioned by the ethics committee of the Iran National Science Foundation.

### Extraction Method

Genomic DNA extraction from peripheral blood samples of both patients and controls was performed using the salting-out method. Following extraction, the concentration and quality of the DNA were meticulously evaluated employing a NanoDrop ND-1000 spectrophotometer at 260 and 280 nm wavelengths. DNA samples with A260/A280 ratios exceeding 1.7 were systematically selected for subsequent analyses. Preserved at −20°C, aliquots of the DNA samples were retained for potential future investigations.

Specific polymerase chain reaction (PCR) primers, integral to the amplification of genomic DNA, were judiciously designed and validated through scrutiny of the single-nucleotide polymorphisms (SNPs) database (dbSNP 129; http://www.ncbi.nlm.nih.gov/projects/SNP/ ) and the BLAST Web site ( http://blast.ncbi.nlm.nih.gov/Blast.cgi ) (see Table 1 for details). Table 2 encapsulates the procedural details wherein genomic DNA underwent amplification by PCR following a predefined program.

**Table 1:** The sequence of the primers and PCR–RFLP product characteristics

Primer name	Sequence	PCR product length (bp)	Digested fragments (bp)
Bsm1-F	GCAACCAAGACTACAAGTACCGCGTCA	845	194 and 651
Bsm1-R	TTTTCTCCCTCTTCTCACCTCTAACCA		
Apa1-F	CTGGCACTGACTCTGGCTCT	634	150 and 484
Apa1-R	GGGCTCACCTGAAGAAGCCT		

Abbreviations: bp, base pair; PCR, polymerase chain reaction; RFLP, restriction fragment length polymorphism.

**Table 2:** PCR program

	Bsm1	Apa1
Cycles	30X	30X
Initial denaturation	94°C	95°C
Time	4 min	4 min
Denaturation	94°C	95°C
Time	1 min	1 min
Annealing	66°C	65°C
Time	1 h 30 min	1 min
Extension	72°C	72°C
Time	1 min	1 min
Final extension	72°C	72°C
Time	7 min	5 min

Abbreviation: PCR, polymerase chain reaction.

For the genotyping of the two polymorphisms under investigation, restriction fragment length polymorphism (RFLP) analysis was employed. Each PCR product underwent digestion with the appropriate restriction endonuclease, adhering to the manufacturer's specifications (Macrogene, Iran). The resulting digested products were separated through electrophoresis on 2% agarose gels and subsequently visualized via ethidium bromide staining under ultraviolet (UV) light. In the context of Bsm1 and Apa1, enzymatically cleaved alleles were denoted by “b” and “a,” respectively, while the undigested alleles were designated as “B” and “A.” The fidelity of RFLP results was rigorously confirmed through the sequencing of randomly selected PCR products.

### Statistical Analysis

The statistical analysis of the acquired data was performed using SPSS Version 18. Utilizing a 2 × 2 table, the data underwent rigorous examination through conditional logistic regression, with subsequent computation of 95% confidence intervals (CIs). For intergroup comparisons, the chi-square test and independent *t* -test were deliberately selected. Significance levels were established at *p* -values less than 0.05, denoting statistical significance.

## Results

### Subject's Data

To investigate *VDR* gene polymorphisms, a cohort of 50 breast cancer patients, with an average age of 47.18 ± 14.36 years, was randomly assembled. Furthermore, 50 healthy subjects, average 43.70 ± 14.70 years in age, were included in the study. The age range within the case and control groups spanned from 18 to 77 and 19 to 80 years, respectively. An independent *t* -test indicated no significant difference in the mean age between the two groups ( *p* = 0.23 and *p* < 0.05).

An examination of menopausal status between the case and control groups, conducted through the chi-square test, indicated a nonsignificant frequency distribution ( *p* = 0.17 and *p* < 0.05). Furthermore, an exploration of familial or sporadic status in breast cancer patients revealed no significant association ( *p* = 0.82).

### Genotyping and Statistics

Table 3 presents the allelic frequencies observed within the respective populations.

**Table 3:** Bsm1 and Apa1 polymorphisms and breast cancer

SNP	Group	Genotypes, *n* (%)			Major allele frequency	Minor allele frequency	*p* -Value	OR (95% CI)	Result
		BB	Bb	bb					
Bsm1	Breast cancer ( *n* = 50)	12 (24)	21 (42)	17 (34)	0.66	0.34	0.50	0.91 (0.40–2.10)	No
	Control group ( *n* = 50)	8 (16)	26 (52)	16 (32)	0.68	0.32			
		**AA**	**Aa**	**aa**					
Apa1	Breast cancer ( *n* = 50)	23 (46)	22 (44.9)	5 (10.2)	0.90	0.10	0.82	1.23 (0.34–4.32)	No
	Control group ( *n* = 50)	20 (39)	24 (49)	6 (12.2)	0.88	0.12			

Abbreviations: CI, confidence interval; OR, odds ratio; SNP, single-nucleotide polymorphism.

### Bsm1 Polymorphism and Breast Cancer Risk

As delineated in Table 3 , an analysis of Bsm1 polymorphisms indicated a distribution of genotypes within the studied cohorts. Specifically, the BB genotype was identified in 24% of cases compared with 16% in controls, the Bb genotype manifested in 42% of patients in contrast to 52% in healthy subjects, and the bb genotype was observed in 34% of cases versus 32% in controls. Subsequent application of the chi-square test revealed no statistically significant disparity between cases and controls ( *p* = 0.14, odds ratio [OR] = 0.91, 95% CI 0.40–2.10).

### Apa1 Polymorphism and Breast Cancer Risk

Classifying women based on their genotype, three distinct groups emerged: AA, Aa, and aa. The chi-square test delineated that the AA genotype was prevalent in 46% of cases compared with 39% in controls, the Aa genotype exhibited frequencies of 44.9% in cases and 49% in controls, while the aa genotype constituted 10.2% of cases versus 12.2% of controls. Consequently, no discernible association between Apa1 polymorphisms and breast cancer risk was evident ( *p* = 0.82, OR = 1.23, 95% CI 0.34–4.32) ( Table 3 ).

### Association Haplotypes and Breast Cancer

Haplotype analysis unveiled that, in terms of their association with breast cancer, two specific haplotypes exhibited significantly higher likelihoods than other prevalent haplotypes. Specifically, the BAT and bAT haplotypes were identified as being linked to an elevated risk of breast cancer. The ORs for the BAT and bAT haplotypes were determined as 2.18 (95% CI 1.35–5.06) and 3.37 (95% CI 1.85–13.26), respectively. In contrast, none of the remaining haplotypes (Bat, baT, BaT, bat, and bAt) demonstrated a statistically significant association with breast cancer risk in this particular population.

## Discussion

Breast cancer, constituting a significant global health concern, represents 9% of the worldwide cancer burden. Prevalence varies markedly, with low-risk regions such as Japan or China reporting 1 in 8 to 1 in 16 cases, while high-risk areas such as Western Europe or North America demonstrate rates of 1 in 4. 34 35 Emerging evidence underscores the regulatory impact of this malignancy on angiogenesis. 36 37 Significantly, an increased intake of vitamin D and higher serum concentrations of its metabolites are associated with a reduced risk of breast cancer. Discovered in 1919 by Edward Mellanby, 38 and later characterized by Norman in 1969, vitamin D plays a multifaceted role beyond skeletal health, influencing cellular differentiation and proliferation. The metabolite 1,25-dihydroxycholecalciferol (1,25(OH)2D3), derived from vitamin D, has demonstrated the capacity to suppress cancer cell proliferation. 39 40 41 Numerous studies have revealed an inverse correlation between vitamin D serum levels and breast cancer risk, 9 42 often associating the effects of vitamin D with the nuclear VDR. 29 The expression of VDR in diverse human tissues, including breast, prostate, bone, monocytes, T and B lymphocytes, gut, and keratinocytes, has expanded the potential therapeutic applications of vitamin D. 43 44 Within the normal mammary gland, the expression of VDR is discernible in epithelial, stromal, and immune cells, with regulatory processes primarily occurring within the epithelial compartment during hormonal fluctuations associated with puberty and pregnancy. 45

Epidemiological studies have reported polymorphisms in the *VDR* gene, such as Fok1, Bsm1, Apa1, and Taq1, associated with breast cancer incidence and risk. 29

Recent investigations have predominantly focused on elucidating the link between VDR polymorphisms and cancer risk, spanning various malignancies including skin, colon, ovarian, bladder, prostate, and breast cancers. Notably, certain studies suggest that the association of VDR genotype polymorphisms with cancer risk may be contingent upon other factors, such as sun exposure. 46 The predominant focus of molecular epidemiological studies in recent years, encompassing both case–control and nested case–control designs, has been on women, aiming to explore the correlation between VDR polymorphisms and the risk of breast cancer. Numerous investigations have delved into the association between the aforementioned polymorphisms, specifically Bsm1 and Apa1, and their relationship to breast cancer. Notably, Apa1 has been implicated in several studies as a potential contributor to an elevated risk of breast cancer development. However, it is imperative to acknowledge that findings regarding the association of VDR variants, including Apa1, with breast cancer have exhibited variability across different studies. 47 48 49 50 Guy et al reported an increased nearly twofold risk for the bb genotype in 2004. 50 51 An additional investigation reported that the Bsm1 polymorphism exhibits linkage disequilibrium with the polyA tail sequence in the 3′ UTR and concurrently identified a noteworthy association between the bb genotype and an increased susceptibility to breast cancer. 52 Ruggiero et al's findings indicated a lack of statistically significant differences in the distribution of the Bsm1 polymorphism between the case and control groups. 52 Nevertheless, within the metastatic cancer subgroup, there was a twofold higher prevalence of the bb genotype compared with the control group, and the proportion of women with the BB genotype and metastases was half that observed in the control group. 10 53

Conversely, an alternative investigation revealed no substantial association between the Bsm1 polymorphism and the risk of breast cancer. It is imperative to acknowledge, however, that this study featured a limited number of cases exclusively of Turkish descent. In contrast, three additional studies involving Taiwanese women, Latinas, and Caucasian women identified an association between the BB genotype and an augmented risk of breast cancer, contradicting the outcomes of the previously mentioned study. 7 54 Hou et al found that the Bsm1 B allele was associated with an increased risk of breast cancer. 53 54

According to the findings of prior investigations, apart from the influence of VDR polymorphisms, the concentration of vitamin D is contingent upon various factors, including seasonal variations (e.g., reduced sunlight exposure during winter months), geographical latitude (with higher UV levels in cities near the equator), skin pigmentation, and cloud cover. Disparities in vitamin D levels among individuals exposed to sunlight underscore the necessity for genetic adaptations to uphold optimal physiological functioning. 25 As a result, differences in results can be attributed to the factors mentioned earlier.

## Conclusion

In summary, the findings of the current investigation suggest that the two examined polymorphisms (Bsm1 and Apa1) within the *VDR* gene may not exhibit a discernible association with breast cancer risk in Iranian women. However, the nuanced nature of such genetic relationships necessitates further comprehensive studies to substantiate these outcomes and illuminate the underlying mechanistic intricacies.

The outcomes of our inquiry into Bsm1 and Apa1, coupled with an expanding body of evidence affirming the protective role of adequate vitamin D levels against breast cancer risk, underscore the substantive role of vitamin D as a significant mediator in the context of breast cancer susceptibility. Consequently, the VDR emerges as a pivotal target meriting consideration in strategies aimed at breast cancer prevention.
